# Exceptional Chromosomal Evolution and Cryptic Speciation of Blind Mole Rats *Nannospalax leucodon* *(Spalacinae*, *Rodentia*) from South-Eastern Europe

**DOI:** 10.3390/genes8110292

**Published:** 2017-10-25

**Authors:** Ivo Savić, Duško Ćirović, Vanja Bugarski-Stanojević

**Affiliations:** 1 Biological Faculty, University of Belgrade, 11000 Belgrade, Serbia; ir_savic@yahoo.com (I.S.); dcirovic@bio.bg.ac.rs (D.Ć.); 2 Institute for Biological Research “Siniša Stanković“, University of Belgrade, 11060 Belgrade, Serbia

**Keywords:** karyotype evolution, chromosomal rearrangements, speciation, cryptic species, cytotypes, chromosomal forms, fossorial, underground

## Abstract

Mole rats are exclusively subterranean and highly specialized rodents. Their long lifespans, remarkable anti-cancer mechanisms, and various distinctive adaptive features make them a useful research model. Moreover, opposing convergence of morphological traits, they have developed extremely high karyotype variability. Thus, 74 chromosomal forms have been described so far and new ones are being revealed continuously. These evolved during the process of rapid radiation and occur in different biogeographical regions. During research into their reproductive biology we have already provided substantial evidence for species-level separation of these taxa. Here, we review diverse chromosomal forms of the lesser blind mole rat, Mediterranean *Nannospalax leucodon*, distributed in South-eastern Europe, their karyotype records, biogeography, origin, and phylogeny from our extensive research. In the light of new data from molecular genetic studies, we question some former valuations and propose a cryptospecies rank for seven reproductively isolated chromosomal forms with sympatric and parapatric distribution and clear ecogeographical discrepances in their habitats, as well as new experimental and theoretical methods for understanding the courses of speciation of these unique fossorial mammals.

## 1. Introduction

With more than 2000 described species, rodents are the most abundant mammalian order, with 42% of its total species diversity [1]. Molecular phylogenetics and cytogenetics has enabled continuous growth in the number of newly discovered species, often revealing morphologically similar but genetically and/or karyotypically divergent cryptic species [2]. Chromosomal rearrangements (CRs) are frequent among rodents, especially in the two most species-rich families, Cricetidae and Muridae [3]. However, there is no default rate of karyotype evolution. While considerable chromosomal conservation was found in certain taxa (castorimorph and anomaluromorph rodents), some karyotypes of the myomorphs were highly reorganized [4,5,6,7,8,9]. Similarly, two genera of the Eurasian blind mole rat (BMRs), subfamily Spalacinae (Gray, 1821) ([1,10,11,12,13,14,15] and others): the greater BMR genus *Spalax* (Guldenstaedt, 1770) and the lesser BMR genus *Nannospalax* (Palmer 1903), have opposed rates of the karyotype change, i.e., highly conserved chromosomes and extensive karyotype variability, respectively. Nowadays many studies reach beyond standard research models, such as the mouse (*Mus*) and the rat (*Rattus*), in order to trace the evolution of crucial characters. Distinctive in many ways, BMRs have become a valuable research object, particularly because of their remarkable resistance to cancer and their longevity [16,17,18,19,20], besides other distinguishing features. These include tolerance to hypercapnia and hypoxia [20,21,22,23,24], a specific lipid profile [25] splice variants of heparanase unique in mammals, as well as particular expression patterns for p53 [26,27], circadian rhythms [28] sensory research [21]. In addition, cytogenetic analysis has revealed a distinguishing karyotype evolution [11,29,30,31]. 

Even though they are appreciated as a research object, BMRs are seriously endangered in their natural habitat, primarily because of an inappropriate conservation status, resulting from an unresolved taxonomy with an unrecognized species [1]. In the International Union for Conservation of Nature (IUCN) Red List of Threatened Species [32], only three species of BMRs are acknowledged and categorized as Data Deficient (DD). Many populations and species are therefore under serious threat, particularly due to the loss and fragmentation of their natural habitats [33,34]. 

**Figure 1:**
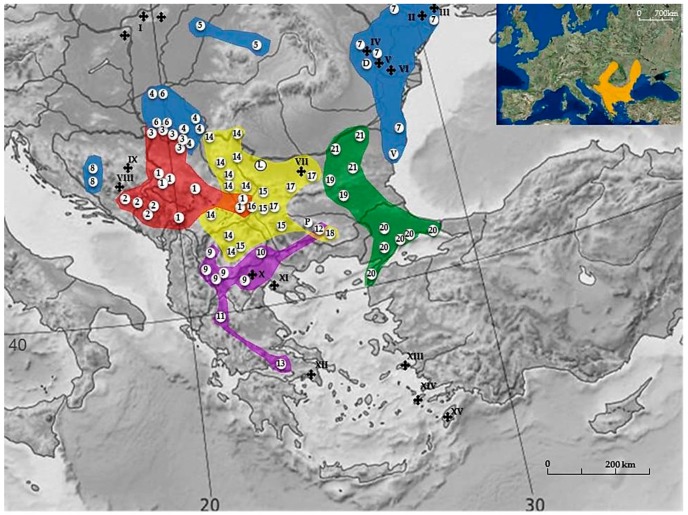
Geographic distribution of *Nannospalax leucodon* chromosomal forms (CF) from South-eastern Europe (reproduced with permission from [29], Figure 2, adapted). Red—the Yugoslav branch; yellow—the Serbicus branch; blue–the North Balkan subsidiary branch; violet–the South Balkan subsidiary branch; green—the East Balkan branch. Small map: *N. leucodon* distribution area from International Union for Conservation of Nature (IUCN) Red List of Threatened Species. For symbols see Table 1.

Eurasian BMRs are typical residents of grassy steppes, hills, and mountain-meadows of the Eastern Mediterranean region, including Eastern and South-eastern Europe, Western Asia, and Northern Africa (Figure 1). They range in altitude from below sea level to an elevation of 2600 m above sea level (a.s.l.) [1,35,36] and also inhabit cultivated areas, sparse woodlands, steppes, and mountain slopes, but do not occur in dense forests and marshy areas [10,13,35,37,38,39,40]. Spalacines are herbivores, eating mostly roots, tubers, rhizomes, and a little grass and grain. With subcutaneous vestigial eyes restricted to photoperiod perception, most of their lifetime is confined to underground tunnels [35]. They are chisel-tooth diggers, using their lower incisors for burrowing and the lower jaw as a shovel. Due to solitary, territorial, and aggressive behavior, their distribution is additionally fragmented [40]. The many adaptations necessary for living underground in cylindrical tunnels have defined their phenotype and obscured their actual phylogenetic relations. Thus, convergent morphology on the one hand and the intensive chromosomal speciation on the other tangle *Spalacinae* systematics and divide taxonomists since they were first discovered. 

### 1.1. Phylogeny and Systematics 

The widely accepted classification [1] groups subfamily Spalacinae in the family Spalacidae, superfamily Muroidea, together with subfamily Myospalacinae (Lilljeborg, 1866), African mole rats Tachyoryctinae (Miller and Gidley, 1918), and Oriental bamboo rats Rhizomyinae (Winge, 1887), as reviewed in [29]. Despite the general convergence of morphological traits, members of the subfamily Spalacinae can be successfully distinguished morphologically from other subfamilies [41].

It was hypothesized earlier that the subterranean BMRs originated from a muroid-cricetoid stock in Asia Minor, or nearby, in the Lower Miocene (now Oligocene times) about 30–40 Mya, and adaptively radiated underground in the Balkans, towards the territories of Germany, Austria, Poland, steppic Russia, and the Middle East, as well as extending southwards to North Africa [35]. They have remained restricted to their current distributional range throughout their evolutionary history. Moreover, its area is being continually reduced, so the territories of present day Germany, Austria, Poland, Western and Central Hungary, Southern Greece, and the Aegean islands are now abandoned [35] (Figure 1). With changes of habitat in certain regions, especially recently due to anthropogenic effects, whole populations have disappeared (North-eastern Greece, some parts of Vojvodina, etc.) [42].

Numerous verifications of the presence of BMRs in the Balkans and Anatolia, throughout the entire Pleistocene [29], dating back to the lower Miocene [43], were recently updated. The genus *Prospalax* was placed in the family Anomalomyidae [44,45,46]. New data show that fossil records of the subfamily Spalacinae can be traced to the Late Oligocene of South-eastern Europe [44,47,48], but the group is probably much older. Currently, only four fossil genera are recognized: *Vetuspalax* [47]; *Debruijnia* [43]; *Heramys* [49] and *Pliospalax* (Kormos, 1932) = *Sinapospalax* (Sarıca and Sen, 2003) [44,47]. The oldest Spalacinae recorded so far is *Vetusspalax progressus* from the late Oligocene of South-eastern Europe, Banovići, Bosnia and Herzegovina (Figure 1) [47].

### 1.2. Speciation

Taxonomy below the subfamily level follows two different concepts: a mono-generic model ([1,35,40] and others) and a two-generic model of the large and small-bodied BMRs, based on morphological and karyological characteristics [10,11,12,13,41]. The taxonomic distinctiveness of these two genera and their monophyletic background is supported by recent molecular evidence [15,42,50]. Though highly similar, the greater BMR genus *Spalax* (Guldenstaedt, 1770) can be distinguished from the lesser BMR genus *Nannospalax* (Palmer, 1903) [12] by substantial morphological differences [10], which are used for fragmentary fossil material as well [47]. Moreover, these morphological characteristics evidently correlate with their different cytogenetic strategies [15]. 

#### 1.2.1. The Greater BMR Genus *Spalax*

This genus includes large-bodied species with karyotypes characterized by the slow rate of chromosomal changes, a high diploid chromosomal number (2n), and a fundamental chromosomal number (NF) (2n = 62/NF = 124, with the single exception of *S. microphthalmus* 2n = 60/NF = 120) and all bi-armed autosomes. These species have been recognized so far by their external, cranial, and dental characters [14]. Interestingly, even with massive geographical barriers between morphologically and/or cytogenetically differentiated *Spalax* species, they share similar morphology and identical 2n and NF with proportionately smaller interspecific differences in karyotypic structure [35]. This indicates that the 62 chromosomal forms (CFs) of blind mole rats had wider ranges in the past and that their karyotypes are relatively stable [51]. Six *Spalax* species are acknowledged: *S. graecus* (Nehring, 1898), *S. arenarius* (Reshetnik, 1938), *S. microphthalmus* (Güldenstaedt, 1770), *S. giganteus* (Nehring, 1898), *S. uralensis* (Tiflov and Usov, 1939), and *S. zemni* (Erxleben, 1777) [1]. Separation of additional species from *S. graecus* in Romania, namely *S. antiquus* (Méhely, 1909) and *S. istricus* (Méhely, 1909), has been proposed based on mitochondrial DNA (mtDNA) sequences and detailed anatomical comparisons [52].

#### 1.2.2. The Lesser BMR Genus *Nannospalax*

The genus *Nannospalax* (*Spalax typhlus leucodon* Nordmann, 1840. Type loc. Near Odessa, Ukraine) consists of small-bodied species with karyotypes characterized by lower diploid numbers with many acrocentric chromosomes and by a proliferating chromosomal speciation observed in the extant species [10,11,29]. Moreover, among the 26 genera of Palaearctic mammals, this genus has the highest karyotype variability [6]. Species and populations of *Nannospalax* exhibit significant 2n and NF diversity, but it is still not clear which events shaped the extant karyotypes. 

Species with low mobility generally have genetically more isolated populations [51] and consequently many cryptic species with a specific karyotype set, as observed in fossorial rodents. Indeed, rapid chromosomal evolution within and between populations and species are most commonly explained as specific adaptations to the underground way of life, low dispersal rates, fragmentation of the distribution range, and isolation of individual populations [11,13,35,53]. This arouses an important question regarding the mechanisms responsible for the completely different strategies in chromosomal evolution in *Spalax* and *Nannospalax* [15,50,52], as they share highly similar morphology, life style, and ecology. 

The proliferating chromosomal speciation of the lesser BMR has brought confusion since the earliest discoveries. *Spalax* (= *Nannospalax*) *leucodon* was first described for a specimen from the Caucasus 2n = 48 [54], then for one female from Bulgaria 2n = 54 [55]. Detailed analysis of karyotypes from Deliblatska Peščara and Vlasina Lake, Serbia, suggested that two forms separated by the River Danube may exist [4]. The complex question of the taxonomy and the courses of speciation of this genus then became highly attractive, but using morphological characters as the basis for classification was not helpful. Therefore, many authors struggled to solve the problem through karyotype analysis. Four allopatric/parapatric chromosomal races with 2n = 52, 54, 58, and 60 were discovered in Israel [53]. Data on chromosomes of almost all extant mole rats species have been collected [11] and extensive research was carried out in South-eastern Europe in subsequent years [29]. 

The number of species acknowledged in the lesser BMR is disparate and ranges from a single one [1,56] to fourteen species in Europe [29]. Most authors recognize three *Nannospalax* species (=morphospecies/superspecies). Thus, *N. leucodon* (Nordmann, 1840), the Mediterranean BMR, inhabits South-eastern Europe; *N. xanthodon* (Nordmann, 1845) (synonym: *N. nehringi* (Satunin, 1898), the Anatolian BMR, inhabits Transcaucasia, most of Turkish Anatolia, and certain East Aegean islands; *N. ehrenbergi* (Nehring, 1898), the Israeli BMR, inhabits South-eastern Anatolia in Turkey, Iraq, Syria, Lebanon, Israel, Jordan, and Egypt. Several authors have joined *N. xanthodon* and *N. leucodon* into *N. leucodon* superspecies (e.g., [39,57]) or a separate genus *Mesospalax* [14]. Anatolia can be considered the core area of differentiation processes in chromosomal evolution within BMRs [31,39,58,59], as all three *Nannospalax* morphospecies were recorded in this region. *N. leucodon* is presumably restricted to the Turkish part of Thrace and its occurrence in the western parts of Anatolia is not certain [40,60]. However, distinguishing between *N. xanthodon* and *N. ehrenbergi* may be difficult even with the use of both morphological and chromosomal characters [61].

Each of these three *Nannospalax* species is most probably a monophyletic collection of closely related and morphologically similar but actively speciating populations at different stages of this process. They putatively evolved through CRs, alternatively entitled cytotypes, chromosomal forms/races, sibling species, good biological species, cryptic species, or evolutionary significant units (ESU’s; Moritz, 1994) [1,4,5,12,14,31,36,40,50,53,62,63,64,65].

A chromosomal race was defined as a group of geographically contiguous or recently separated populations, which share the same chromosome complement by descent, using the common shrew *Sorex araneus* as an example [66]. The authors further proposed that geographically isolated populations with the same karyotype should be merged into the same race, only if there is proof that they have separated recently and share all their chromosomes by common ancestry. This view was adjusted to the BMRs [29]. Populations with similar karyotypes differing particularly in the number of chromosomal arms or even in 2n were exceptionally included in the same race = CF, because variability observed within one CF may be an artefact of comparisons of findings published in different reports. For CFs with proved reproductive isolation, specific ecological preferences, and wide biogeographical distribution, we find it appropriate for now to use the term cryptic species or cryptospecies. This corresponds to two or more morphologically similar evolutionary lineages that were erroneously classified and hidden under one species name (see [67]). 

## 2. Mediterranean BMR *N. leucodon* Cryptospecies

The taxonomy, speciation mechanisms, origin, and distribution patterns of Spalacinae from South-eastern Europe have been studied from different aspects. However, many questions, mostly concerning taxonomy and phylogenetic interrelationships between different CF (or cryptospecies), are still open. On the basis of the above mentioned, it was established that the territory of former Yugoslavia was settled by a total of eight different species of the genus *Spalax* (= *Nannospalax*) [68,69]. The research was later continued [5,68,69,70] and all available cytogenetic, morphometric, craniometric, paleontological, zoogeographic, and biogeographic data summarized [29]. Six different 2n forms were recorded: 38, 48, 52, 54, 56, and 58, with no evident intrapopulation karyotype polymorphism (Table S1). However, the morphology of the autosomes within 2n = 54 and 56 forms, between different population groups, often varied greatly, causing altered NF and NFa values. At the same time, it was shown that certain karyotype forms inhabit wide distributional areas. 

### 2.1. Phylogenetic Dendrogram 

Considering common characters in karyotype structure within some karyotype forms (e.g., a very small pair of metacentrics; the first two to three pairs of the largest subacrocentrics; or the two smallest pairs of submetacentric autosomes), morpho- and craniometric differences, dissimilarities in habitat, natural barriers (large forest complexes, wide bodies of water, humid soil, rocky deserts, different altitudes), and evidence for reproductive isolation, a hypothetical dendrogram with phylogenetic relationships and the speciation courses between all documented karyotypically different forms was constructed [29,71]. It was hypothesized that during the process of allopatric speciation, a variety of karyotypes emerged as a result of adaptation to different environments. Therefore, from a common initial ancestor (which probably had 2n~60 and a high number of acrocentric chromosomes) four basic groups of CFs have diverged: the Yugoslav branch, the Central Balkan branch, the Serbicus branch, and the East Balkan branch (the numbers of CFs in the text correspond to Figure 1, Table 1, Tables S1 and S2).

I **The Yugoslav Branch** (NF = 82–90) is characterized by the presence of a very small pair of metacentric autosomes.
  1. *montanoserbicus* (Savić and Soldatović, 1974) 2n = 56; NF = 82; with 15–16 pairs of small acrocentrics is probably a relic of an ancestor originally settled in the Central Balkan region. Today its populations occupy a wide discontinuous area of island-type isolates of preglacial Mid-Balkan Mountains (the Rhodope Mt.—Dinaride in Hercegovina and Montenegro), over 700 m a.s.l. It is possible that the area of this CF spreads to the territory of the Rhodopes in neighboring Bulgaria as well. The forms *hercegovinensis* and *syrmiensis* probably originate from it [29]. They share an identical 2n and NF, but with significant dissimilarities in the morphology of some autosomal groups. There are no data regarding reproductive isolation between them. Nevertheless, considering the morpho and craniometric discrepancies, the geographical distance between their distributional areas and the biogeographic differences among their habitats, they could be considered as a biologically separate species—cryptospecies.
  2. *hercegovinensis* (Mehely, 1909) 2n = 54; NF = 90 was described as a separate form (species) [68] recorded in the Dinaric Mountains in Montenegro and Hercegovina.
  3. *syrmiensis* (Mehely, 1909) 2n = 54; NF = 90 is a group of populations with different chromosomal morphology from *hercegovinensis*. They occupy the plains of Srem and the right bank of the rivers Sava and Danube from the entry of the river Drina into the Sava and the Great Morava into the Danube. A slight discrepancy in karyotype structure (see Table S1) has been recognized in one population (Banovo Brdo, Belgrade).
II **The Central Balkan Branch** is characterized by very similar NF values, divided into two subsidiaries: the North Balkan branch (*hungaricus*, *transsylvanicus*, *leucodon*, *montanosyrmiensis*, and *monticola*) and the South Balkan branch (*hellenicus*, *thracius*, *strumiciensis*, *makedonicus*, and *epiroticus*). This group diverged very early with 2n almost 60 for *hellenicus*, *epiroticus*, and *leucodon*, all characterized with fourteen pairs of acrocentric autosomes. The CF *leucodon* drifted to the northern part of the Balkan Peninsula and generated the northern branch—CFs *hungaricus*, *transsylvanicus*, *montanosyrmiensis*, and *monticola* with variable 2n = 48–54, but almost identical NF, namely 84 or 86. The Makedonian branch diverged from the *epirus* CF, while the CFs *strumiciensis* and *thracius* separated from the initial branch somewhat later.
**IIa**  **North Balkan subsidiary branch.** The existence of reproductive isolation between some CFs has been proved.
  4. *hungaricus* (Nehring, 1898) 2n = 48; NF = 84 inhabits a large area of Pannonian lowland in Bačka, Banat, and a narrow belt in northern Serbia, where it overlaps with *syrmiensis* on the slopes of Mt. Avala.
  5. *transsylvanicus* (Mehely, 1909) 2n = 50; NF = 84 is distributed in North-western Romania [62] and Eastern Hungary [33]. It was described as a possible subspecies of *N. hungaricus* [29] because of the highly similar karyotype.
  6. *montanosyrmiensis* (Savić and Soldatović, 1974) 2n = 54; NF = 86 is a relict species present on Fruška Gora, where two populations with a difference in the Y chromosome exist, and additionally in Kelebia on the Serbian/Hungarian border [42].
  7. *leucodon* (Nordmann, 1840) 2n = 56; NF = 84 is distributed in Moldavia, Dobrudzha, Odessa and South-western Ukraine [11]. There is a certain chromosomal similarity with *dobrudzha* CF, so a recent hypothetical dispersal of *N. leucodon* into Moldova and Southern Ukraine from the southwest was suggested. Nearby on the Black Sea coast, Eastern Bulgaria, *varna* CF (2n = 52, NFa = 76, NF = 80) was reported but only from description of the locality [72].
  8. *monticola* (Nehring, 1898) is a relict species 2n = 54; NF = 84 that occupies the western border of the Mediterranean BMR range, i.e., Kupreško Polje, Bosnia and Herzegovina. The area probably spreads to the central parts of the Dinaric massif. Although the karyotype is similar to that of *montanosyrmiensis*, morpho- and craniometrics diverge on a greater scale.
**IIb**  **South Balkan subsidiary branch** (2n = 54, 56 and 58; NF = 84 and 88). They inhabit the South-eastern and far Southern parts of the Balkan Peninsula. According to karyotype similarities and morpho- and craniometric characters, several forms could belong to this CF:
  9. *makedonicus* (Savić and Soldatović, 1974) 2n = 52; NF = 86 inhabits the South-western Balkan Peninsula including Western Makedonia, North-western Greece, and probably spreading into neighboring Albania.
  10. *strumiciensis* (Savić and Soldatović, 1974) 2n = 54; NF = 88 occurs in Dabilja, Strumica Valley, Macedonia (FYROM).
  11. *epiroticus* (Savić, 1982) 2n = 56; NF = 84 is found in Lefkothea, Epirus, Greece.
  12. *thracius* (Savić, 1982) 2n = 56; NF = 88 inhabits Novo Selo on the Thracian plain, Bulgaria.
  13. *hellenicus* (Mehely, 1909) 2n = 58; NF = 88 was recorded in Parnas, Greece.
  According to their morphological characters several more CFs were described as *thermacius* (Hinton, 1920) (= *strumiciensis*), *insularis* (Thomas, 1917), *thessalicus* (Ondrias, 1966), and *peloponnesiacus* (Ondrias, 1966) [29].
III **The Serbicus Branch** has a very high NF of 90–98. The first CF to develop was the Sofia-East population with 11 pairs of acrocentric autosomes. CFs *ovchepolensis* and *serbicus* were formed later, as well as the *tranensis* CF, represented by only one population and possibly *rhodopiensis* CF, also represented by a single population in Dobrostan, Bulgaria, although this form digresses slightly from other CFs of this branch.
  14. *serbicus* (Mehely) 2n = 54; NF = 98 includes several CFs that are reproductively isolated from neighboring CFs. They inhabit valleys of Eastern Serbia, Northern Makedonia, Kosovo, and South-western Bulgaria. The suggestion that morpho- and craniometric characters are similar to *monticola*, *hungaricus*, and *montanosyrmiensis*, because of a common ancestor, should be further explored. Some authors recognize the highly similar CF *lom* in two isolated areas in North-western and South-western Bulgaria [72].
  15. *ovchepolensis* (Savić and Soldatović, 1974) 2n = 54; NF = 94 is found at Ovče Polje, Makedonia (FYROM).
  16. *tranensis* (Peshev, 1981) 2n = 54; NF = 96 was recorded in Tran, Bulgaria.
  17. *sofiensis* (Peshev, 1983) 2n = 56; NF = 90 was registerd in Cherven Briag, Bulgaria.
  18. *rhodopiensis* (Peshev, 1981) 2n = 54; NF = 92 occurs in Dobrostan, Bulgaria and should be analyzed in greater detail regarding karyotype structure [72].
IV **East Balkan Branch.** Contrary to pronounced 2n differences, the NF values vary within narrow limits of 74 to 78 and therefore it was suggested that Robertsonian fusions of acrocentric autosomes were mostly responsible for the karyotype transformations. No crossbreeding experiments have been done in this group.
  19. *turcicus* (Mehely, 1909) 2n = 56; NF = 78 has seventeen pairs of acrocentric autosomes and was the first to diverge from the basic branch. Now it probably represents the oldest existing form and inhabits the Lower Thrace lowlands.
  Out of the same group, two CFs were formed in Bulgaria — *srebarna* and *kozarevets*, with nine and eight pairs of acrocentric autosomes, respectively.
  20. *bulgaricus* (Peshev, 1981) 2n = 46 and 48; NF = 76 consists of two populations with different karyotypes.
  21. *srebarnensis* (Peshev, 1981) 2n = 48; NF = 78 is found in Russe, Targoviste, and Silistra regions in North-east Bulgaria.

Populations from the western part of Asia Minor, the island of Lesbos, and probably other islands in the Aegean Sea, as well as being designated as *N. nehringi* (Satunin, 1898) 2n = 38; NF = 74. (*N. n. anatolicus* Mehely, 1909), were later shown to belong to the Anatolian BMR species *N. xanthodon* CF *anatolicus* [14,40]. 

### 2.2. Natural Hybrids

Some of these CFs live sympatrically, i.e., their areas touch or partially overlap. Investigations in the bordering zones of populations with different karyotypes revealed no cases with natural hybrid karyotypes [29]. The closest contact was noticed between *hungaricu*s and *syrmiensis* on the slopes of Mt. Avala, where they live sympatrically in the same area (more precisely, in the same meadow) and between *hungaricu*s and *montanosyrmiensis* in Kelebia-Subotička peščara [46]. Great differences in chromosome morphology could be the most probable reason for this reproductive isolation. The CF *syrmiensis* is also territorially linked to the *montanosyrmiensis* form, but their areas diverge vertically. Likewise, no animals with hybrid karyotypes have been registered either. Two different CFs were observed on other mountains. Thus, *montanoserbicus* occupies the higher altitudes of Mt. Kopaonik and the Balkan Mountain range, but in the lower areas and foothills the *serbicus* form was very densely settled. Even without any natural obstacles, not a single hybrid was found in the contact zones. Also, the parapatric CFs, *makedonicus*, *serbicus*, and *ovchepolensis*, with boundaries in central Makedonia, similarly showed no natural hybrids. Thus, the conclusion is that the separate CFs are probably different biological species (cryptospecies), reproductively isolated for a long time.

Remarkably, despite a variety of karyotypes in BMRs, hybrids between individual chromosomal forms have been found only sporadically over the entire distributional area. Hybrids are also apparently absent or very infrequent in Anatolia [39,57,73,74,75], with only three 2n = 49 probable cases located in Central-eastern Anatolia [76]. Hybridization is commonly described only for Israel [53,77], with lower fitness compared to their parents [78]. 

### 2.3. Experimental Crossbreeding

Experimental crossbreeding results [29] greatly contributed to the conclusion that karyotype changes lead to reproductive isolation. When males and females belonging to the same CF were paired, they mated with resulting embryos. Moreover, when animals from the same CF but from geographically distant populations were paired, the result was always positive (Table 2). However, although pairing individuals with different CFs was followed by mating in most cases, no embryos were formed. Pre and post copulation reproductive isolation between different CFs demonstrated by experimental crossbreeding was confirmed by artificial insemination performed in similar combinations. Embryos developed only when the same CFs were combined. Chromosome preparations made from embryo fibroblast cultures showed no differences between their karyotypes and those of their parents. These experiments did not include all recorded *N. leucodon* CFs. Instead, importance was given to marginally sympatric forms, with touching or overlapping distributional areas, or sites separated by natural barriers/different altitudes.

## 3. Chromosomal Rearrangements in *Nannospalax leucodon*

The extensive karyotype variation in *Nannospalax* arises from numerous and still insufficiently identified chromosomal changes, because studies using banding techniques or molecular cytogenetic methods have been rather rare [79,80,81,82]. Mechanisms of chromosomal evolution already recorded in this group are Robertsonian rearrangements (fusions and fissions), additions/deletions of C-heterochromatin, pericentric inversions, centromeric shifts, euchromatin deletions, positional changes of the nucleolar organizing regions (NOR) sites, missing whole chromosomes, or supernumerary B chromosomes [29,53,60,79,83]. Most confirmations about mechanisms of karyotype evolution have hitherto been obtained for Israeli BMR species and cytotypes.

In general, Robertsonian rearrangements are considered to be the major mechanism of 2n chromosomes changes in *Nannospalax*. Processes of divergence were probably peripatric with Robertsonian changes fixed in small isolated marginal populations, though the direction of these alterations is a matter of long lasting debate. It is difficult to decide which of the following hypotheses is correct. The fusion hypothesis assumes that, during the karyotype evolution of *N. leucodon*, Robertsonian rearrangements most probably acted in the direction of a decrease in the number of acrocentric autosomes and 2n [29,60]. Similarly, some authors considered chromosomal fusion as the major force of karyotype evolution in BMRs [81,84,85]. *The fission hypothesis* suggests a reverse tendency for increasing 2n through Robertsonian fissions in both Turkey and Israel, by fission of metacentrics to form acrocentric chromosomes, as the major initial mechanism of chromosomal evolution in BMRs [58,83]. During this process the number of acrocentrics increases, while changes in the NF derive from centromeric shifts. The ancestral *Spalacine* karyotype was 2n = 38, increasing progressively in different lineages [58,83].

According to analysis of ten Anatolian CFs, 2n = 60C is the ancestral CF and 2n = 38 and 2n = 60K are secondary ancestral CFs [81]. Therefore, Robertsonian fusions have the main role in chromosomal evolution of BMRs in Turkey, while Robertsonian fissions and pericentric inversions/deletion are minor forces in their chromosomal evolution [79,81]. Which factor causes these rearrangements is still not conclusively known and there is a clear necessity for detailed chromosomal studies using both differential staining techniques and molecular cytogenetic methods that, up till now, have been very limited. 

G-banded chromosomes of *N.* (= *Spalax*) *leucodon* have been described only from two distant populations. Namely, Mt. Bistra, North-western Makedonia (2n = 52, NF = 86) [86], and *N. xanthodon* (labelled as *leucodon*) from Malatya, Turkey (2n = 60, NF = 78) [79]. C-banding distribution of NORs was accomplished only for the Malatya population [79]. 

Notwithstanding the enormous chromosomal differentiation observed among *N. leucodon* species, only a few could be used to distinguish between them. Distinctive markers, i.e., the chromosomal changes observed between the recognized taxa, included two or three noticeably large subtelocentric autosomal pairs characterizing the karyotype of most populations of the Mediterranean BMR, *N. leucodon*, from South-eastern Europe with certain exceptions (*varna*, *bulgaricus*, and *srebarnensis* CF) [29]. 

### The Fusion or Fission Hypothesis?

It is difficult to define any universal chromosomal changes that could explain the course of speciation of currently recognized taxa of BMRs. As stated above, Robertsonian rearrangements, fixed in small isolated marginal populations, are considered to be the major mechanism of 2n chromosomes changes in *Nannospalax*. However, the direction of these changes is a matter of a long lasting debate. 

Chromosomal speciation and adaptive radiation of BMRs in Asia Minor and the Middle East were correlated with increased ecological stress [38,57,58]. This association was established, firstly due to the possibility that fissions of metacentric chromosomes largely increase haplotype diversity. This may further elevate population adaptation to climatic stress. Secondly, there is the notion that species and races with the highest 2n from the entire Spalacinae distributional area occupy the most xeric regions and those with the lowest 2n live in mesic habitats in the center of their range. A number of studies (Section 4) have indicated that the speciation course of the Israeli BMR *N. ehrenbergi* from lower to higher 2n as it moves southwards to a more arid and warmer habitat is highly credible. However, generalization of these events to other lineages of BMRs, widely distributed in Anatolia and South-eastern Europe, is not sound. The majority of *N. leucodon* CFs have not been thoroughly studied, especially by molecular methods. Furthermore, the complex biogeographical history of the Balkan Peninsula [87], as well as new paleontological data [44,47,48], do not allow such universalization of speciation events. More detailed genetic research including extant and extinct samples, combined with molecular cytogenetic methods, are necessary to explain karyotype evolution in this group. 

Elevation to species level of three *N. xanthodon* CFs was recommended based on genetic separation [88]. Although the studied sample included ten individuals with 2n = 60, one with 2n = 58, and two with 2n = 40, the authors argue that the basal position of the CF with the lowest 2n of 40 in the inferred phylogenetic tree (rooted with *N. ehrenbergi* from Diyarbakir locality, 2n = 52 or 56) strongly supports the fission hypothesis. However, other molecular phylogenetic studies showed that the populations with lower 2n did not hold basal positions, but rather appeared in the internal branches [14,50,65,89]. Several additional investigations indicated chromosomal fusion as the major force for karyotype evolution in BMRs [81,84,85]. A phylogenetic dendrogram based on G- and C-banding techniques showed that populations with 2n = 60 were the ancestors of all CFs [81]. The opposite direction of Robertsonian rearrangements and a decrease in the number of acrocentric autosomes, together with 2n [29,60], has acquired several more confirmations. Namely, monophyly of two major lineages of extant BMRs, the genera *Spalax* (2n = 60–62) and *Nannospalax* (2n = 38–60), was confirmed by comprehensive research [10,15,50,52], so the most probable scenario is that their common ancestor had a karyotype with a high 2n (60) involving mostly acrocentric autosomes. It was observed that the ancestral form of chromosomally diversified species is most probably the one with the largest distribution [7]. Thus, populations with the highest 2n of 60 from both *Spalax* and *Nannospalax* genera are widely distributed. For example, the *N. xanthodon* cytotype 2n = 60 inhabits almost all climatic zones in Anatolia [73,90]. 

As for recent representatives, on the basis of phylogenetic dendrogram studies of fossil *Spalacinae*, it was established that several parallel courses of speciation exist at the same time, as well as certain side branches with blind endings [29]. Finally, it seems that the direction of *Nannospalax* karyotype evolution is more complicated and variable. The evolution courses of the three morphospecies (= species groups) *N. leucodon*, *N. xanthodon*, and *N. ehrenbergi*, could be completely divergent, i.e., the fusion hypothesis may be valid for *N. leucodon* and some *N. xanthodon* representatives and the fission hypothesis for *N. ehrenbergi*. 

## 4. Molecular Research in the Subfamily Spalacinae

In general, the majority of molecular studies have explored the Israeli *N. ehrenbergi* species complex [14,50,57,58,65,91], while Mediterranean BMRs (*N. leucodon* CFs) have received only sporadic attention. Thus, the overall phylogenetic pattern in all extant BMRs is far from resolved.

Recent speciation events and slight genetic changes were reported in an isozyme study on seven Spalacinae species from both genera [51]. The results were in agreement with the karyological classification: I *N. nehringi* (TfA, HbB); II *N. leucodon* (TfB, HbC); III *S. microphthalmus* (TfC, HbA); IV *S. graecus* (TfB, HbA/HbB); *S. polonicus* (TfB, HbB); *S. arenarius* (TfB, HbB); and *S. giganteus* (TfB, HbB) [10].

The Major Histocompatibility Complex (MHC), highly polymorphic in both classes of polypeptides in *N. ehrenbergi*, showed low polymorphism in four Balkan CFs: *N. syrmiensis*, *N. montanosyrmiensis*, *N. hungaricus*, and *N. makedonicus* [92]. The estimation that *N. leucodon* CFs might have very low genetic diversity should be considered with caution due to limited sampling (three out of the four examined species inhabit steppe habitats of the Sub Pannonian hilly and mountain foot areas, some of them even sympatrically), difficulties were reported with mouse probe hybridizations with *Spalax* DNA, etc. 

Allozyme diversity studies support the environmental selection hypothesis of genetic diversity among *N. ehrenbergi* CFs from Turkey, Israel, and Egypt [58]. Genetic distances (*D*) ranged from 0.001 to 0.269, with the highest value between ancestor Turkish and descendant Israeli and Egyptian species. It was estimated that climatic selection in Turkey appeared to be the major diversity factor in both speciation and adaptation. Accordingly, mtDNA diversity in *N. ehrenbergi* was found to be significantly correlated with the climate, pathogens, and different molecular and physiological factors [93]. Gene flow and introgression seemed to play a minor role, while natural selection at the macro- and microgeographic levels appeared to be the major differentiating factor. mtDNA diversity in the youngest species *(*2n = 60) was higher than in the oldest species pair (2n = 52 and 54).

In agreement with the above-mentioned findings, a study of 1140 bp cyt *b* sequences in 53 individuals of all three morphospecies of the lesser mole rat [14] pointed to division of the genus *Nannospalax* into two subgenera *Nannospalax s.s. (N. ehrenbergi*) and *Mesospalax (N. xanthodon* and *N. leucodon)* [12]. However, sixteen individuals of *N. leucodon* from this sample, namely *N. hungaricus*, *N. serbicus*, *N. makedonicus*, and *N. hercegovinensis*, were designated indirectly without karyotyping, by presuming their 2n/NF from localities recorded 40–50 years earlier [29]. There was a mean distance of 5 km between the new and previously documented localities. On the contrary, the karyotypes and DNA sequences of *N. xanthodon* and *N. ehrenbergi* specimens were analyzed completely and confirmed. The authors found that among the three morphospecies, genetic diversity was lowest in *N. leucodon* (2.4% ± 0.3%), highest in *N. xanthodon* (8.8% ± 0.7%), and intermediate in *N. ehrenbergi* (5.0% ± 0.5%). However, while *N. leucodon* comprises 25 CFs, *N. xanthodon* 29 CFs, and *N. ehrenbergi* 20 CFs [31,61], only four CFs of *N. leucodon* were included (2n = 48, 52, and 54) and, as expected, the lowest genetic diversity was recorded among them. The highest diversity was in *N. xanthodon* (the sample 2n ranged from 38 to 60), while genetic diversity among *N. ehrenbergi* (2n = 52, 54, 58, and 60) was intermediate. Therefore, conclusions regarding the origin and further evolutionary scenario deduced from these results should be taken with reserve, especially in the light of the newest data [47,48]. 

The most comprehensive phylogenetic analysis of five mtDNA sequences (12S ribosomal RNA (rRNA), transfer RNA (tRNA)-Val, 16S rRNA, tRNA-Leu (UUR), NADH dehydrogenase subunit 1 (NADH1), tRNA-Ile, 3742 bp in total) in 41 samples from 35 different populations of BMRs [50] indicated the highest rates of heterogeneity according to the Maximum likelihood phylogram between *Spalax/Nannospalax* 0.1577. Inside the genus *Spalax*, heterogeneity rate was 0.039; between the two superspecies, *N. leucodon* and *N. xanthodon*, 0.0623, but much higher (0.1066) between these two superspecies on one side and *N. ehrenbergi* at the other. For *N. leucodon* superspecies, comparable values were: 0.0447 for *montanosyrmiensis* to *srebarnensis*; 0.0255 *srebarnensis* to *hungaricus*; and 0.0113 *hungaricus* to *transsylvanicus*. In *N. ehrenbergi* superspecies, the values between species were slightly higher: 0.0397 for *galili* to *golani*; and 0.0361 *juadei* to *carmeli*. Inside *N. xanthodon* superspecies the range was from 0.0172 to 0.056. 

Comparison of 1140 bp mtDNA sequences of four individuals of *S. graecus* with 43 sequences from *N. leucodon*, *xanthodon* and *ehrenbergi* samples from GenBank [15], provided noticeably higher pairwise Kimura two-parameter genetic divergences among the genera *Spalax* and *Nannospalax*, than between three *Nannospalax* species. These results confirmed the taxonomic distinctiveness of the two genera. 

As the evolution of a particular gene is not necessarily identical with the evolution of the species [94], above results of mtDNA cyt *b* gene analysis could serve only as an initial step in untangling the complex evolutionary history of the BMRs. 

Microsatellite (MS) markers analysis of the twelve populations of *S. ehrenbergi* superspecies [95] revealed positive correlation between MS diversity and aridity stress. Natural selection appears adaptively to determine MS evolution in *Spalax* regionally across the distributional area. Very low gene flow was reported between species pairs, except for one population of *N. carmeli* (2*n* = 58) located near the hybrid zone between it and *S. golani* (2*n* = 54). This analysis confirmed the earlier described pattern [58,89,96] of northern (older) and southern (younger) species pairs, which represent different stages of evolutionary divergence. 

## 5. Cryptic Speciation in *N. leucodon*

It is well known that chromosomal number and morphology are general characteristics of the species of one genus, or even genera from the same family. Thus, karyotype polymorphism between them is not a common phenomenon. The highest rates of karyotype variability are found in muroid rodents, canids, gibbons, and equids. Nevertheless, in each of them there are taxa with completely opposite rates of chromosomal change: e.g., the Sciuridae family among rodents, apes among primates, and rhinoceroses among perissodactyls [8]. Environmental effects, total mutation rates, population size, mobile elements, and retroviruses could contribute to this distinction [8]. The “Court Jester” evolution model, which promotes the effects of Quaternary climatic change on speciation in mammals, was recently proposed for Spalacinae species [50]. Paleobiological studies indicate that large-time scale patterns of biodiversity are driven by the physical environment, including geological and tectonic events, landscape, food supply, or climate. Besides strong support for the majority of branching events on the tree, the absence of support in a few instances indicates that network-like evolution could exist in BMRs [50]. 

Similarly to *Nannospalax*, there are other well-documented examples of mammalian species complexes with clear chromosomal separation of the species, i.e., groups of chromosomal forms (e.g., *Rhogeessa tumida*, *Ellobius tancrei*, and *Nannomys minutoides*) [97]. The most diversified are the 72 known chromosomal “races” of the common shrew, *Sorex araneus* [98] and 97 chromosomal “populations” in the house mouse, *Mus musculus* [99]. Chromosomal forms in these two species were formed recently and most studies showed no signal of molecular diversification [98,99]. In comparison, *Microtus arvalis* (2n = 46) has two chromosomal races (Western European *arvalis* and Eastern Asian *obscurus*) that are considered subspecies because they are not reproductively isolated, despite the chromosomal and genetic differentiation (demonstrated by Fluorescence In Situ Hybridization (FISH) analysis). Therefore, they are classified into the superspecies complex *Microtus arvalis* s.l. 

There are multiple instances that provide support for the hypothesis that some of the 74 described *Nannospalax* CFs [31,61] represent valid cryptic species. Besides three morphospecies, *N. leucodon*, *N. xanthodon*, and *N. ehrenbergi*, the only CFs acknowledged in the literature as separate species are four Israeli BMRs, *Spalax* (= *Nannospalax) galili*, *S. golani*, *S. carmeli*, and *S. judaei* [36,96,100]. These species represent young, closely related allospecies in the early stages of speciation, i.e., as the evolutionary youngest, occupying different climatic regimes. Nevertheless, phylogenetic analysis did not confirm separation of the last two species, *S. carmeli* and *S. judaei* [65,96,100], similarly to earlier findings [89]. Moreover, hybrids were frequently reported only in Israel [53,77], with lower fitness compared to their parents [78]. 

Recently it was suggested that four BMR species inhabit Anatolia—*N. ehrenbergi* in the southeast, *N. nehringi* in the east, *N. xanthodon* in the west, and *N. labaumei* in central Anatolia [101]. Moreover, four CFs of *N. xanthodon* 2n = 36, 38, 40, 52, should be treated as valid biological species [75,102]. 

Here we recommend cryptospecies rank for seven reproductively isolated *N. leucodon* CFs (Table 2, Table S2) with sympatric and parapatric distribution: *Nannospalax montanoserbicus* (Savić and Soldatović, 1974), *Nannospalax syrmiensis* (Méhely, 1909), *Nannospalax makedonicus* (Savić and Soldatović, 1974), *Nannospalax hungaricus* (Nehring, 1898), *Nannospalax montanosyrmiensis* (Savić and Soldatović, 1974), *Nannospalax monticola* (Nehring, 1898), and *Nannospalax serbicus* (Méhely, 1909), according to the following criteria: reproductive isolation (absence of hybrids), sympatric/parapatric distribution with ecogeographic differences, morphological modifications, and genetic distance.

### 5.1. Reproductive Isolation

In the early stages of speciation, phenotypic, karyotypic, and genotypic evolution rates may progress independently [7]. The fact that karyotype differences influence speciation throughout the appearance of reproductive isolation was recorded long ago in two types of morphologically similar fruit flies, previously treated as one species [103]. For such cases, the term sibling, i.e., sister species was introduced, describing the presence of reproductive isolation as the basic criterion for raising a natural population, or group of populations, to the species rank [104]. More than 40 new cryptic species were recorded in the Palaearctic and 24 in Europe [7]. Some CRs can cause fertility problems or sterility in hybrids, acting as genetic barriers to gene flow between populations with fixed chromosomal differences [105,106]. In two parapatric populations connected by symmetric gene flow, chromosome changes can delay the fixation of favorable alleles and allow incompatibilities to accumulate [107]. Populations will become increasingly differentiated until speciation is complete. Genic and nongenic mechanisms can act together in speciation, as proposed a long time ago [108]. It was also observed that groups with higher rates of chromosomal changes have greater speciation rates [109]. The significant role of CRs in the process of speciation was later confirmed in other organisms [3,29,36,110]. 

In the case of mole-rats, reproductive isolation, together with the adaptation of diverse chromosomal forms to different ecological conditions [57,83,111], leads to complete detention in the gene flow. During chromosomal speciation, changes like Robertsonian fusions occur in peripheral populations [106] proceeding slowly to reproductive isolation from the main population. Since genetic discrepancy still remains low, a small hybrid zone may be retained between them. However, genetic and morphological differences accumulate and recently separated populations disperse into new areas or habitats [106]. Poor movability, solitary, and territorial and aggressive behavior have certainly contributed to spatial isolation and the appearance of divergent speciation of karyotype forms leading gradually to their complete reproductive isolation [60,68]. 

The experimental hybridization method has been successfully applied to refine unclear taxonomic affiliation of various forms [112,113]. It provides important information about the degree of divergence of closely related species and allows the mechanisms of reproductive isolation to be studied. Such experiments, together with artificial insemination and the absence of hybrid individuals in their natural habitats, have proved complete reproductive isolation and elucidated post- and/or pre copulatory isolation mechanisms. As mentioned above, natural hybrids are apparently absent or very infrequent in Anatolia [39,65,73,74] but are commonly described only in Israel with lower fitness than their parents [53,77]. 

### 5.2. Sympatric/Parapatric Distribution

Numerous described cryptic species have sympatric distribution, providing an important indirect confirmation of complete reproductive isolation of these distinctly derived groups [114]. Genetic differentiation of allopatric populations, however, might have contributed to local adaptation or genetic drift. An indicative feature of chromosomal changes in the genus *Nannospalax* is the parapatric or allopatric pattern of distribution of populations with a specifically changed karyotype. There are a few exceptions in *N. leucodon*, as some sympatrically distributed cryptospecies exist [29]. 

Models of chromosomal speciation do not postulate that all speciation events are due to CRs. Populations are likely to diverge genetically in allopatry and, under certain conditions, they also have the opportunity to accumulate CRs [110,115]. In order to explore if cryptic species are more frequent in allopatric or sympatric habitats, chromosomal speciation in 41 pairs of sister species was tested in the two most species-rich rodent families, *Cricetidae* and *Muridae*, to reveal a direct role of CRs in speciation [3]. About 30% of sister species had an identical karyotype, and they were not randomly distributed but were more common for allopatric sister species than for sympatric ones. This study indicated that, after secondary contact, it is more likely that karyotypically diverged species will remain distinct than genetically diverged ones. This is because hetero-karyotypes are expected to be less fit than homo-karyotypes [110].

The results obtained for phylogenetic relationships of the karyotypic forms and possible evolution paths were confirmed by data concerning their biogeographic distribution [60]. Besides its geographical position between Europe, Asia, and Africa, the Balkan Peninsula is considered to have the most complicated relief due to diverse geomorphology and frequent changes in global ecological conditions [116].

### 5.3. Morphological/Physiological Modifications

Despite the extensive chromosomal variability, morphological and physiological modifications are not easily noticeable in BMRs, although some do exist [29,71]. It is also a common assumption that speciation of the most cryptic species is a recent event, so morphological characters or other diagnosable features have not had time to evolve. This may be true for some taxa, such as coccolithophores, but studies of bonefish amphipods and copepods show ancient divergences among cryptic species [114]. Even though chromosomal changes may induce morphological and physiological modifications, a disparate number of chromosomes (2n) does not necessarily alter the phenotype. Two morphologically very similar deer species, Indian and Chinese muntjacs, with 2n = 6 and 46, respectively, provide an extreme example [117]. 

A number of studies have explored the morphological characters of *N. leucodon* CFs. ([21,71,118] and others). The results of craniometric analysis were correlated with 12 CFs from twenty different populations and directions of their migrations and speciation in the Balkan Peninsula BMRs were discussed [71]. Generally, specific phenotypic characteristics should be explored in Balkan CFs in greater detail. For example, there are some animals (insects, frogs, and fish) that communicate non-visually through sound, vibration, pheromones, or electrical signals, possibly hiding cryptic species, because changes in these types of signals may lead to reproductive isolation that is not morphological [114]. Therefore, studies of non-visual communication might contribute to explanations of reproductive isolation in BMRs. 

### 5.4. Genetic Distances

To infer the amount of genetic change developing together with chromosomal rearrangements and phylogenetic estimates in such comparative studies, representative sampling is crucial. Therefore, the majority of CFs of each morphospecies should be equally included. Although the species rank of *N. leucodon* CFs was questioned, giving low cyt *b* genetic divergences [14,50], these studies were based on comparison of only four CFs, which is a small proportion of the 25 CFs described in total [31]. Furthermore, pairwise comparisons of genetic distances within the Mediterranean BMRs were of a similar scale as those among Israeli BMRs [14,50] and some *Spalax* species [50], which are already acknowledged as separate species. For example, genetic diversity within the *serbicus/makedonicus* lineage was 1.6% ± 0.2%; within the two major monophyletic lineages of the *N. ehrenbergi* morphospecies from Israel: *galili*/*golani* (1.4% ± 0.2%) and *carmeli/judaei* (1.4% ± 0.3%). 

According to an older hypothesis, subterranean and fossorial mammals are generally characterized by significantly lower genetic diversity than other above-ground mammal species [119]. DNA-DNA hybridization studies indicated the relative number of interspecific nucleotide substitutions between four species of *N. ehrenbergi* to be 0% to 5%, which suggests that adaptive chromosomal speciation does not have to be accompanied by major genomic changes [91]. Instead, it may happen with minor genomic changes in these animals.

There are almost 20 CFs of the Mediterranean BMRs *N. leucodon* reported that should be involved in future molecular genetic research, including other genes beside cyt *b*, to obtain more realistic phylogeographical patterns. New sampling and comparison with older karyological data is required to confirm their presence and distribution.

### 5.5. Further Perspectives

Chromosome fissions and fusions, common mechanisms in karyotype evolution, represent illegitimate events that occur during meiosis, which are associated with changes in chromosome number [120]. Many of the breakpoint sites are connected to the formation of acrocentric chromosomes in some species and metacentric in others, e.g., between the domestic dog and the red fox [8]. 

Other types of more complex change detected by modern methods of molecular cytogenetics can be used in evolutionary studies. Structural differences (accidental crossing over between homologous segments on non-homologous chromosomes) may result in interchromosomal rearrangements and may be initiated by a chromosomal inversion within one of the segments. The breakpoint sites are usually located near telomeres and centromeres [8]. These structural changes do not necessarily produce phenotypic effects (e.g., geographically separate populations of *Mus musculus*), but they may induce reproductive isolation [121]. The reasons for differences in the rates of these illegitimate recombinations in different species during their evolution are still unknown. 

Comparison of different directions in karyotype evolution of the conservative *Spalax* and the highly variable *Nannospalax* could provide answers to these questions. Exploring these discrepancies might reveal a possible connection to more fragile chromosomal regions like conserved or rare fragile sites. Lately, an effort has been made to explain the mechanisms of chromosomal instability at evolutionarily conserved fragile sites and their correlation with cancer [121]. It was discovered that the chromosomal breakpoints occurring in constitutionally balanced CRs in the human karyotype have a non-random distribution. Interestingly, highly significant associations of rare fragile sites were found with both evolutionary breakpoints and tandem repeats, with important implications for their role in chromosomal instability and therefore genome evolution [122]. The authors provided clear evidence for the existence of fragile chromosomal regions that are prone to reorganization and have been conserved in different lineages during evolution.

Molecular cytogenetic techniques, like FISH painting using whole chromosome probes, extend cytogenetic investigations of karyotype evolution, revealing a wide range of interchromosomal translocations. Cross-species chromosome painting has been used for comparative cytogenetic studies in rodents, identifying conserved blocks of chromosome homology between species and discovering combinations that reveal their evolutionary relationships [123]. However, *Mus musculus* chromosomes are highly rearranged and hence problematic to some extent for use in comparative studies in preference to those derived from species with conserved genomes (e.g., [124,125]). Accordingly, flow-sorted painting probes, isolated from the naked mole-rats, *Heterocephalus glaber* (2n = 60), revealed probable fixations of CRs favored by environmental factors and/or their specific social structure [126]. The relatively limited resolution of whole chromosome probes does not allow detection of smaller rearrangements and intra-chromosomal changes, like inversions. Therefore, improved FISH-banding approaches, such as multicolor banding (MCB) [127,128,129], would be the method of choice for further research on chromosome evolution in BMRs and are essential for correct interpretation of genomic sequencing data in future studies. Consequently, it is necessary to develop specific BMR chromosome probes to characterize chromosomal breakpoints, map the observed CRs and measure their functional, genomic consequences in both non-variable *Spalax* and highly variable *Nannospalax*. 

We suggested unofficial taxonomic rank cryptospecies for seven *N. leucodon* CFs, which exist as separate biological species. The actual species status is yet to be proved with further studies. About twenty described CFs of the Mediterranean BMRs should be involved in future molecular genetic research, including other genes besides cyt *b*, to obtain more accurate phylogeographical patterns. New sampling and comparison with older karyological data are required to confirm their presence and distribution. Also, to identify and describe species-level diversity, it is crucial to use an integrative taxonomy based on evolutionary history, morphology, behavior and genetics. Comparing samples from extinct species with extant ones will allow reconstruction of the mechanisms of evolutionary and ecological processes that lead to divergence and reproductive isolation in the absence of morphological differentiation.

## Figures and Tables

**Table:** (**a**)

	CF	2n	NF	Sampling Localities	Branches [29]
1	*montanoserbicus*	56	82	Tara, Zlatibor, Čajetina, Čigota, Kopaonik, Vlasina-Klisura, Vlasina-Sokolovo, Čakor.	I
2	*hercegovinensis*	54	90	Durmitor, Zelengora, Maglić, Čemerno, Neretva, Gvozd, Njegovuđa.	
3	*syrmiensis*	54	90	Višnjica, B. brdo, Košutnjak, Avala, Jajinci, Smederevo-Udovice, Bogatić (Mačva), St. Pazova.	
4	*hungaricus*	48	84	Hajdukovo, Šušara, Dolovo, Jajinci, Avala.	IIa
5	*transsylvanicus*	50	84	Jucu, Cluj-Napoca region, Transylvania.	
6	*montanosyrmiensis*	54	86	Stražilovo, Čortanovci, Kelebia.	
7	*leucodon*	56	84	Odessa, Orgeev, Bacau, Perieni, Constanta.	
8	*monticola*	54	84	Kupreško Polje, Šuica, Ljubuša Mt.	
9	*makedonicus*	52	86	Jakupica Mt., Karadžica, Pelagonia, Prilep, Bitolj, Ohrid, lake Vegoritis, Arnissa.	IIb
10	*strumiciensis*	54	88	Strumičko Polje (Strumica Valley).	
11	*epiroticus*	56	84	Lefkothea (Epirus, NW Greece).	
12	*thracius*	56	88	Novo Selo, Plovdiv (Bulgaria).	
13	*hellenicus*	58	88	Levadia (Southern Greece).	
14	*serbicus*	54	98	Biskuplje, Rogljevo, Resavska Cave, Ram (Đerdap), Resava, Kladovo, Rtanj Mt., Niš, Pirot, Priština, Katlanovo, T.Veles.	III
15	*ovchepolensis*	54	94	Ovče Polje (Eastern Makedonia).	
16	*tranensis*	54	96	Tran (Western Bulgaria).	
17	*sofiensis*	56	90	Sofia-East, Cherven Briag (Bulgaria).	
18	*rhodopiensis*	54	92	Dobrostan near Asenovgrad (Bulgaria).	
19	*turcicus*	56	76–78	European Turkey, Lower Thrace.	IV
20	*bulgaricus*	46	76	Kozarevets near Veliko Tarnovo, Sliven region, 370 m a.s.l. (Bulgaria).	
21	*srebarnensis*	48	78	Srebarna, right bank of the River Danube, 80 m a.s.l. (Bulgaria).	
D	*dobrudzha*	54–56	78–84	Dobrudzha (Romania).	
L	*lom*	54	98	Lom (Bulgaria).	
P	*pazardzhik*	54	86	Pazardzhik (Bulgaria).	
V	*varna*	52	80	Varna (Bulgaria).	

**Table:** (**b**)

	Fossil Findings	Locality
I	*N. cf. leucodon*	West and North Hungary
II	*N. macoveii*	Grebenniki
III	*N. odessanus*	Odessa
IV	*N. macoveii*	Berešti
V	*N. macoveii*	Gavonosy (Gabanoasa)
VI	*N. macoveii*	Malušteni
VII	*N. cf. leucodon*	Golema Lisza peschtera
VIII	*N. cf. leucodon*	Marinova Cave
IX	*Vetuspalax progressus*	Banovići (BandH)
X	*Debruijinia kostakii*	Karydia (Greece)
XI	*N. macoveii*	Serrai
XII	*Pliospalax tourkobouniensis*	Tourkobonnia Hill
XIII	*N.cf. nehringi*	Chios
XIV	*N.cf. nehringi*	Kalymnos
XV	*Pliospalax sotirisi*	Maritsa

CF: chromosomal form; 2n: diploid chromosomal number; NF: fundamental chromosomal number; a.s.l: above sea level.

**Table 2:** Experimental crossbreeding results, from [29].

Female CF	Male CF	Mating	Embryos
hungaricus *	hungaricus *	+	+
*hungaricus*	*syrmiensis*	+	−
*hungaricus*	*montanoserbicus*	−	−
*hungaricus*	*montanosyrmiensis*	+	−
*syrmiensis*	*montanosyrmiensis*	+	−
*syrmiensis*	*montanoserbicus*	−	−
*makedonicus*	*montanoserbicus*	+	−
*montanoserbicus*	*serbicus*	+	−
*syrmiensis*	*serbicus*	+	−
*monticola*	*hungaricus*	+	−
*monticola*	*montanosyrmiensis*	+	−
*montanosyrmiensis*	*syrmiensis*	+	−
*syrmiensis*	*hungaricus*	+	−
montanoserbicus *	montanoserbicus *	+	+

* Combinations of the same CF from geographically distant populations.

## Supplementary Materials

The following are available online at www.mdpi.com/2073-4425/8/11/292/s1. Table S1: Karyotype structure of *N. leucodon* chromosomal forms (CF) from South-eastern Europe. M—metacentric, SM—submetacentric, A—acrocentric, SA—subacrocentric; Table S2: Biogeographic distribution of *N. leucodon* chromosomal forms (CF) from South-eastern Europe.
