# Supplementary material for: Exceptional Chromosomal Evolution and Cryptic Speciation of Blind Mole Rats Nannospalax leucodon (Spalacinae, Rodentia) from South-Eastern Europe
Source: Genes (Basel). 2017 Oct 25;8(11):292. doi: 10.3390/genes8110292 (PMC5704205; doi:10.3390/genes8110292)
Supplement: Supplementary file 1 [file genes-08-00292-s001.zip › Table S1 proofred.docx]

|  | Chromosomal form (CF) | 2n | NFa | NF | Metacentrics (M) | Submetacentric (SM) | Subacrocentric (SA) | Acrocentric (A) | X | Y |
| --- | --- | --- | --- | --- | --- | --- | --- | --- | --- | --- |
| 1 | *montanoserbicus* | 56 | 76-78 | 82(80) | 2 pairs (medium and the smallest) | 6 pairs (4 large to medium and 2 small) | 4 large pairs | 15 small pairs | large SM | small A |
| 2 | *hercegoviensis* | 54 | 86 | 90 | 3 pairs (large, medium and the smallest) | 6 pairs (4 large to medium and 2 small) | 8 pairs (first the largest and the rest large to medium) | 9 small pairs | large SM | small A |
| 3 | *syrmiensis* | 54(56) | 86-90 | 90(94) | 3 pairs (large, medium and the smallest) | 9 pairs (7 large to medium and 2 small) | 5 large pairs | 8 small pairs | large SM | small A |
| 4 | *hungaricus* | 48 | 80 | 84 | 4 larger pairs | 8 pairs | 5 (some of them the largest) | 6 the smallest | medium SM | small A |
| 5 | *transsylvanicus* | 50 | 80 | 84 | 4 medium pairs | 7 pairs | 5 pairs (two very large) | 8 pairs | large SM or M | SM or A |
| 6 | *montanosyrmiensis* | 54 | 82 | 86 | 2 medium pairs | 8 pairs | 5 the largest | 11 small pairs | large SM | small A or M |
| 7 | *leucodon* | 56 | 80 | 84 | 2 pairs (different size) | 3 pairs | 8 mostly large pairs (first two the largest) | 14 small pairs | large SM | small A |
| 8 | *monticola* | 54 | 80 | 84 | 2 large pairs | 7 pairs (5 large to medium and 2 small) | 5 large pairs | 12 small pairs | large SM | small A to SA |
| 9 | *makedonicus* | 52 | 82 | 86 | 2 large pairs | 7 pairs (6 large to medium and 1 small) | 7 pairs (large to medium) | 9 small pairs | large M | small A |
| 10 | *strumiciensis* | 54 | 84 | 88 | 4 medium pairs | 6 pairs (4 medium and 2 small) | 6 large pairs | 10 small pairs | large SM | small A |
| 11 | *epiroticus* | 56 | 80 | 84 | 1 medium pair | 6 pairs (4 large to medium and 2 small) | 6 large pairs (first the largest) | 14 small pairs | medium to large SM | small A |
| 12 | *thracius* | 56 | 84 | 88 | 1 medium pair | 7 pairs (5 medium and 2 small) | 7 large pairs | 12 small pairs | large SM | small A to SA |
| 13 | *hellenicus* | 58 | 84 | 88 | 1 medium pair | 7 pairs (5 large to medium and 2 small) | 6 large pairs | 14 small pairs | large M | small A |
| 14 | *serbicus* | 54 | 94 | 98 | 4 pairs (2 large and 2 medium) | 10 pairs (8 large to medium, 2 smaller) | 7 large/medium pairs (first the largest) | 5 small pairs | large M | smaller A to SA |
| 15 | *ovchepolensis* | 54 | 90 | 94 | 4 medium pairs | 8 pairs (6 large to medium and 2 small) | 7 large pairs | 7 pairs all small | large SM | small A |
| 16 | *tranensis* | 54 | 92 | 96 | 4 pairs (medium and small) | 9 pairs (medium and small) | 7 pairs (first two the largest) | 6 small pairs |  |  |
| 17 | *sofiensis* | 56 | 86 | 90 | 2 smaller pairs | 9 pairs (medium and small) | 5 pairs (first two the largest) | 11 small pairs | medium SM | small A |
| 18 | *rhodopiensis* | 54 | 88 | 92 | 1 large pair | 7 pairs (medium and small) | 10 pairs (first two the largest) | 8 small pairs | large SM | not distinguished |
| 19 | *turcicus* | 56 | 72 (74) | 76(78) | 2 medium pairs | 5 pairs (4 medium, 1 small) | 3 pairs (first 2 the largest, one medium) | 17 pairs (3 medium and 14 small) | large SM | small A |
| 20 | *bulgaricus* | 46 | 72 | 76 | 7 pairs (large to medium) | 5 (the first two the largest of the set) | 2 (a large and a medium) | 8 small pairs | medium SM | small ST |
| 21 | *srebarnensis* | 48 | 74 | 78 | 5 pairs (one the smallest) | 7 pairs (the first two the largest of the set) | 2 (a large and a medium) | 9 small pairs | medium SM | not distinguished |
| V | *varna* | 52 | 76 | 80 | 3 pairs (medium/small) | 8 pairs | 2 (a large and a small) | 12 small pairs | large M | small A |
| P | *pazardzhik* | 54 | 82 | 86 | 3 medium pairs | 9 pairs | 3 pairs (2 are the largest) | 11 small pairs | medium SM | small A |
| L | *lom* | 54 | 94 | 98 | 5 pairs (1 large, 4 medium/small) | 11 pairs (medium/small) | 5 pairs (first 2 the largest) | 5 pairs | medium SM | not distinguished |
| D | *dobrudzha* | 54-56 | 74-80 | 78-84 | 13 (12) biarmed (polymorphic) | | 14 (15) acrocentric (polymorphic) | | large SA or SM | small SA |
