# Supplementary material for: Exceptional Chromosomal Evolution and Cryptic Speciation of Blind Mole Rats Nannospalax leucodon (Spalacinae, Rodentia) from South-Eastern Europe
Source: Genes (Basel). 2017 Oct 25;8(11):292. doi: 10.3390/genes8110292 (PMC5704205; doi:10.3390/genes8110292)
Supplement: Supplementary file 1 [file genes-08-00292-s001.zip › Table S2 proofred.docx]

|  | CF and description locality | 2n | NFa | NF | Geographic distribution | Biogeographic division * | Soil types |  |
| --- | --- | --- | --- | --- | --- | --- | --- | --- |
| **1** | ***montanoserbicus***  (Savić et Soldatović, 1974) Zlatibor, Serbia. | 56 | 76-78 | 80-82 | Tara, Zlatibor, Čigota, SW Serbia, Kopaonik, Balkan Mt. system in SE Serbia, Dinaric Mt. system, Prokletije; alt. above 700 m a.s.l. | Xeromontano steppe habitats in preglacial Mid Balkan Mts. (North Dinarides, Rila-Rhodope and the Balkan system). | Good drainage, light mechanical composition, low colloid clay. |  |
| **2** | ***hercegovinensis***  (Méhely, 1909) Ulog-Obrnja, Herzegovina. | 54 | 86 | 90 | The territories of Hercegovina, Čemerno and Neretva, Gvozd Mt. Montenegro. | Xeromontano steppe habitats of the Montenegro/East Bosnian Mountains of the Dinaric system (North Dinarides) | Humus-silicate layers and deluvium with good drainage. |  |
| **3** | ***syrmiensis***  (Méhely, 1909) Stara Pazova, Srem, Serbia. | 54(56) | 86-90 | 90(94) | Belgrade region, Avala Mt. Srem, Mačva. | The steppe habitats of the Pannonian plain and the Ancient-Pannonian shoreline – the lower belt. | Marl sand for the basis and arable steppe. |  |
| **4** | ***hungaricus***  (Nehring, 1898) Dolovo, Southern Banat, Serbia. | 48 | 80 | 84 | The Pannonian lowland of Bačka and Banat and across the Danube in the Northern Serbia; Deliblatska and Subotička peščara; The southern border -Mt. Avala, Jajinci, where it meets the *syrmiensis* CF. | The steppe habitats of the Pannonian Lowland and Ancient-Pannonian coast (vegetational zone of forest-steppes in the Mid-Danube valley, namely the vegetational circle Chrysopogonetalia-danubiale). | Marl sand for the basis and arable steppe. |  |
| **5** | ***transsylvanicus***  (Méhely, 1909) Puszta-Szt.-Miklos, Transylvania, Romania. | 50 | 80 | 84 | North-western Romania and  Eastern Hungary. | Xeromountain-steppe habitats of the Carpathian Mountain System (Transylvania) | / |  |
| **6** | ***montanosyrmiensis***  (Savić et Soldatović, 1974) Fruška Gora Mt., Srem, Serbia. | 54 | 82 | 86 | The foothills of Mt. Fruška Gora, in the southern edge of the Pannonian plain in Serbia and in neighboring regions (Kelebia) in Hungary. | The steppe habitats of Sub-Pannonian hilly and piedmont areas - the brim of Fruška Gora. | Layers of marl. |  |
| **7** | ***leucodon***  (Nordmann, 1840) Odessa,Ukraine. | 56 | 80 | 84 | Odessa region in the SW Ukraine and Orgeev region in Moldova. | Steppe habitats of Pontic lowland (vegetational zone of forest-steppes in the s. str. Lower Danube valley). | / |  |
| **8** | ***monticola***  (Nehring, 1898)  Kupreško polje, Bosnia and Herzegovina. | 54 | 80 | 84 | The Dinaric Mts. in the Western Bosnia and the southern slopes of Mt. Ljubuša in Herzegovina). | The steppe karst fields of the Mid Bosnian Mountains of the Dinaric system (Northern Dinarides). | Deluvial soils with some gravel and relatively deep layer of soil. |  |
| **9** | ***makedonicus***  (Savić et Soldatović, 1974) Mt. Jakupica  Makedonia (FYROM). | 52 | 82 | 86 | Mt. Jakupica, above 2200 m and lower elevations in Pelagonia (from Prilep to Bitola) and in the Lake Ohrid basin, as well as in valleys of northern Greece. | Xeromontano steppe habitats of the preglacial South Balkan Mountains (south Dinarides). | Humus-silicate and deluvium soil types, good drainage. |  |
| **10** | ***strumiciensis***  (Savić et Soldatović, 1974) Dabilja, Strumica Valley, Makedonia (FYROM). | 54 | 84 | 88 | Possibly the area spreads to the Southern and Eastern parts of Makedionia, along the Strumica river, towards the territories of Greece and Bulgaria. | Steppe habitats in border region of the territories of the Makedonian hilly and piedmont area and the Rila-Rhodope mountain system. | Dry arable steppe, molehills visible only in humid periods. |  |
| **11** | ***epiroticus***  (Savić, 1982) Lefkothea, Epirus, Greece. | 56 | 80 | 84 | Epirus rocky semidesert (high altitudes in Makedonia, lower in Pelagonia, Lake Ohrid basin and valleys of northern Greece). | Xeromontano-steppe habitats of the preglacial South Balkan Mountains (the South Dinarides- the territory of Epirus). | Rocky semidesert. |  |
| **12** | ***thracius***  (Savić, 1982)  Novo Selo, the Thracian plain, Bulgaria. | 56 | 84 | 88 | South-Eastern parts of the lower Thrace (probably spreading to Greece and Bulgaria) arable steppe with a deep layer of soil. | Xeromountain-steppe habitats of the Thracian plain (the vegetational province of the Thracian steppes). | Arable steppe. |  |
| **13** | ***hellenicus***  (Méhely, 1909)  Parnas, Greece. | 58 | 84 | 88 | Southern Greece, Beotia, Attika Penninsula. The population occurred on river banks at about 250 m a.s.l. Only two males were examined. | Xeromontano-steppe habitats of the preglacial South Balkan Mountains (the mountains of the southern Greece). | Dry, rocky semidesert. |  |
| **14** | ***serbicus***  (Méhely, 1909)  Pirot, Serbia. | 54 | 94 | 98 | From the Danube near Ram and Djerdap, southwards down the Great Morava valley, the Timok, the South Morava and the Vardar to Veles in Makedonia; Kosovo in the West and up to Pirot to the East. | The steppe habitats of the valleys of Serbia and Makedonia (the Mesian and Makedonian hilly and piedmont area). | Dry lands, well drained, with ingredients of sand and gravel. |  |
| **15** | ***ovchepolensis***  (Savić et Soldatović, 1974) Ovče Polje, Makedonia (FYROM). | 54 | 90 | 94 | Northeastern Makedonia continues northeastward to the Sofia Valley, Bulgaria. | Semidesert steppe lowlands of eastern Makedonia (the Makedonian hilly and piedmont area). | Dry arable steppe, molehills visible only in humid periods. |  |
| **16** | ***tranensis***  (Peshev, 1981) Tran, Bulgaria. | 54 | 92 | 96 | Tran, the upper course of the River Nišava in the westernmost Bulgaria, 700 m a.s.l. Single female examined only. | | / |  |
| **17** | ***sofiensis***  (Peshev, 1983) Cherven Briag, Bulgaria. | 56 | 86 | 90 | The central parts of northern Bulgaria between the Balkan (Stara Planina) Mts. and the River Danube and the Pleven region. East of the range of the *lom* and *ovchepolensis* CF. | | / |  |
| **18** | ***rhodopiensis***  (Peshev, 1981*)*  Dobrostan, Bulgaria. | 54 | 88 | 92 | Dobrostan near Asenovgrad, 1200 m a.s.l., Bulgaria Northern slopes of the Rhodopi Mts. in the southern parts of central Bulgaria. | | / | / |
| **19** | ***turcicus***  (Méhely, 1909)  Makri-Koi Istanbul, Turkey. | 56 | 72-74 | 76-78 | The lower regions of Upper Thrace, Maričko polje, probably spreading to Bulgaria and Greece. | Steppe habitats of the Thracian plain (vegetational province of the Thracian steppes). | Arable steppe with a deep layer of soil. |  |
| **20** | ***bulgaricus***  (Peshev, 1981)  Kozarevets, Bulgaria. | 46 | 72 | 76 | Kozarevets near Veliko Tarnovo, Sliven region, 370 m a.s.l., Bulgaria. Known from the slopes of the Balkan Mts. (Stara Planina) in the eastern parts of the central Bulgaria, east of the range of the *sofiensis* *cf.* | | / |  |
| **21** | ***srebarnensis***  (Peshev 1981) Srebarna, right bank of the River Danube, 80 m a.s.l., Bulgaria. | 48 | 74 | 78 | Russe, Targoviste and Silistra regions in NE Bulgaria. | | / |  |
| **V** | ***varna***  (Peshev, 1983) Varna at the Black Sea coast, Eastern Bulgaria. | 52 | 76 | 80 | The race is known only from the description locality. | | / |  |
| **P** | ***pazardzhik***  (Peshev, 1983) Pazardzhik, central Bulgaria. | 54 | 82 | 86 | Known only from the description locality where two males were examined. | | / |  |
| **L** | ***lom***  (Peshev, 1983) Lom, right bank of the River Danube, North-Western Bulgaria. | 54 | 94 | 98 | Two isolated areas of distribution were recorded in  Western Bulgaria, in NW and SW parts of the country. | | / |  |
| **D** | ***dobrudzha***  (Raicu et al. 1968) Constanta, Dobrudzha,  Romania. | 54-56 | 74-80 | 78-84 | Dobrudzha, Black Sea coast, Moldavia (Perinei,  Bacau) in Romania. | | / |  |

*Biogeographic division: according to Matvejev, S. *Biogeogafija Jugoslavije. Osnovni Principi*; Biološki Institut NRS: Beograd, Serbia, 1961; p*.* 232.
